# Ill-Temper a Symptom of Excessive Meat-Eating

**Published:** 1896-03

**Authors:** 


### Ill-Temper a Symptom of Excessive Meat-Eating.

One deplorable result of excessive meat-eating in England is the ill-temper which is a chronic moral complaint among us. In no country, I believe, is home rendered so unhappy and life made so miserable by the ill-temper of those who are obliged to live together as in England. To everybody who read these lines examples will occur of homes which are rendered quite unnecessarily unhappy, when they might be happy, by the moroseness and rudeness of the head of the family, by the peevishness of the wife, or by the quarreling of the younger members.

If we compare domestic life and manners in England with those of other countries where meat does not form such an integral article of diet, a notable improvement will be remarked. In less meat-eating France urbanity is the rule of the home; in fish and rice-eating Japan harsh words are unknown, and an exquisite politeness to one another prevails even among children who play together in the streets. In Japan I never heard rude, angry words spoken by any but Englishmen. I am strongly of the opinion that the ill-temper of the English is caused in a great measure by too abundant meat dietary combined with a sedentary life. The half-oxidized products of albumen form urates and uric acid, which, circulating in the blood, produce both mental and moral disturbances.—Mrs. Ernest Hart, *in "Diet in Sickness and in Health,"*

